# Supplementary material for: Comprehensive Analysis of the Prognosis and Drug Sensitivity of Differentiation-Related lncRNAs in Papillary Thyroid Cancer
Source: Cancers (Basel). 2022 Mar 7;14(5):1353. doi: 10.3390/cancers14051353 (PMC8909347; doi:10.3390/cancers14051353)
Supplement: Supplementary file 1 [file cancers-14-01353-s001.zip › Figure legend.pdf]

Figure legend

Figure S1. A: PPI network shows the relationship among differentiation-related regulators. B: Pearson correlation analysis of the differentiation-related regulators.

Figure S2. A: Lasso regression of the 5 DR-lncRNAs, the lines represent the DR-lncRNA. B: Cross-validation for tuning the parameter selection in the LASSO regression (λ=5).

Figure S3. The relationship between risk score and immune checkpoints blockade.

Figure S4. GO and KEGG pathway enrichment functional analysis. A: Biological process. B: Cellular component. C: Molecular function. D: Pathway analysis.

Figure S5. The structure of the chosen drugs.

Figure S6. The GSE3360 database to validate the expression of DR-lncRNAs. A: the expression of DR-lncRNAs among PTC, ATC, and normal tissues. ATC: anaplastic thyroid cancer. PTC: papillary thyroid cancer
