# Supplementary material for: Comprehensive Analysis of the Prognosis and Drug Sensitivity of Differentiation-Related lncRNAs in Papillary Thyroid Cancer
Source: Cancers (Basel). 2022 Mar 7;14(5):1353. doi: 10.3390/cancers14051353 (PMC8909347; doi:10.3390/cancers14051353)
Supplement: Supplementary file 1 [file cancers-14-01353-s001.zip › supplementary Table S1.pdf]

Table S1. Quantitative real time PCR primers

**Primer Name**		**Primer Sequence (5′-3′)**
TNRC6C-AS1	Forward	ACTGTGGGCGTGAGCTTCTTT
	Reverse	GCTTCTGCGTCACCTTGTAGTC
DPH6-DT	Forward	GAATGGAAAGATGAGCAATGGG
	Reverse	TGGTCCCAAATGTGAGACGAAT
LINC00900	Forward	CATTCGCTCCTCATAGGGACA
	Reverse	TCTCAGGCTGTGATGGGAATGT
CASC15	Forward	CCAGCAGAATTACCTGAGACGAG
	Reverse	GAAACAGACAATGGAGAACAGCC
GAPDH	Forward	GGAGCGAGATCCCTCCAAAAT
	Reverse	GGCTGTTGTCATACTTCTCATGG
