# Supplementary figures and images for: Comprehensive Analysis of the Prognosis and Drug Sensitivity of Differentiation-Related lncRNAs in Papillary Thyroid Cancer
Source: Cancers (Basel). 2022 Mar 7;14(5):1353. doi: 10.3390/cancers14051353 (PMC8909347; doi:10.3390/cancers14051353)

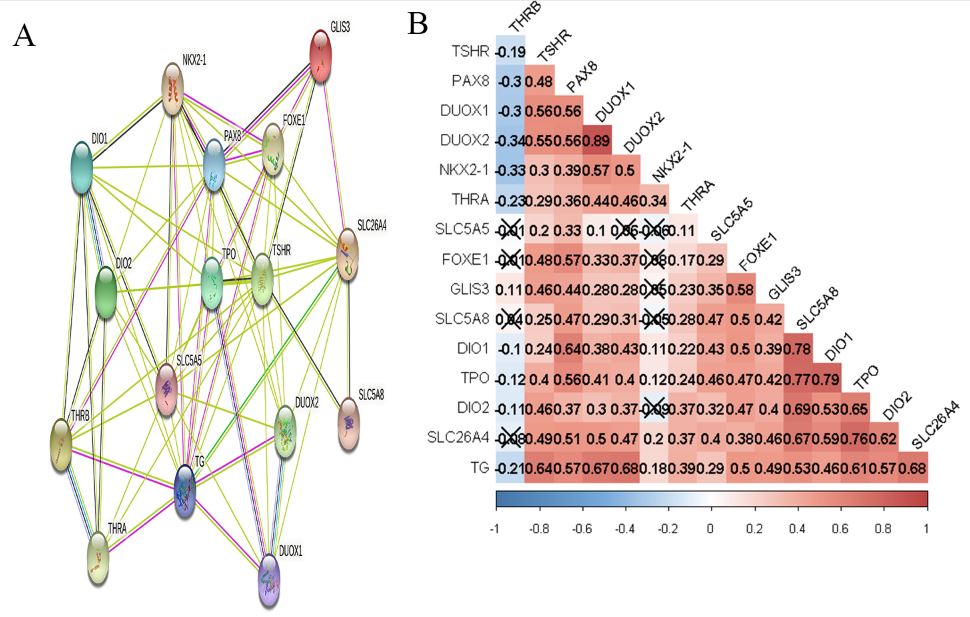

Supplement: Supplementary file 1 [file cancers-14-01353-s001.zip › Figure S1.JPG]

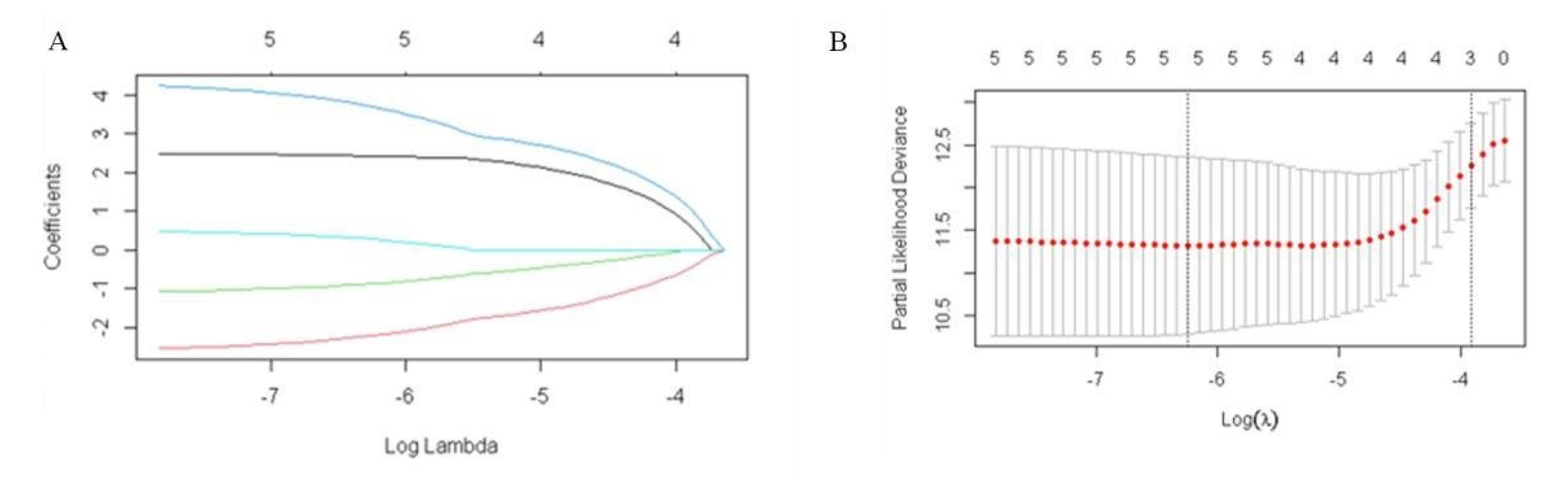

Supplement: Supplementary file 1 [file cancers-14-01353-s001.zip › Figure S2.tif]

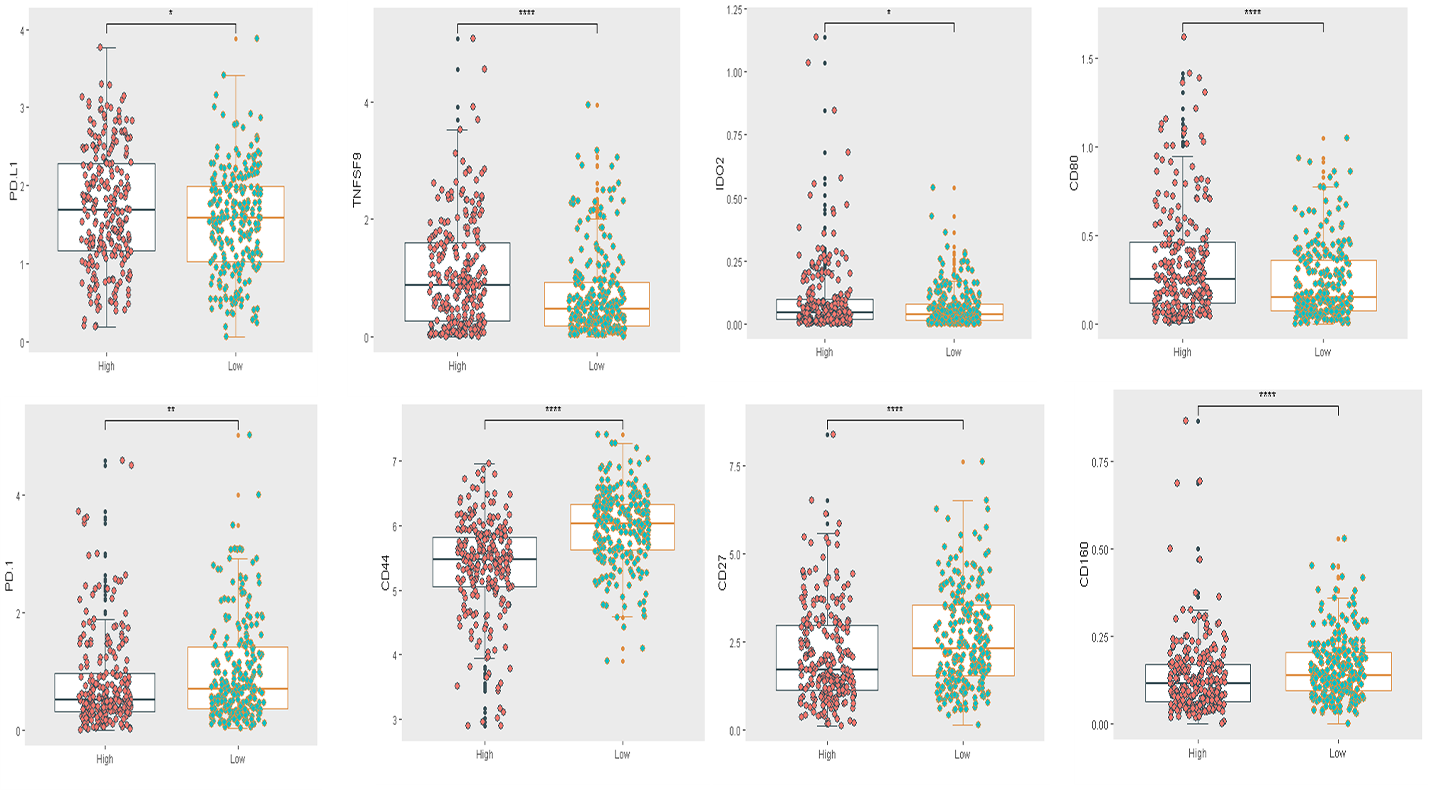

Supplement: Supplementary file 1 [file cancers-14-01353-s001.zip › Figure S3.tif]

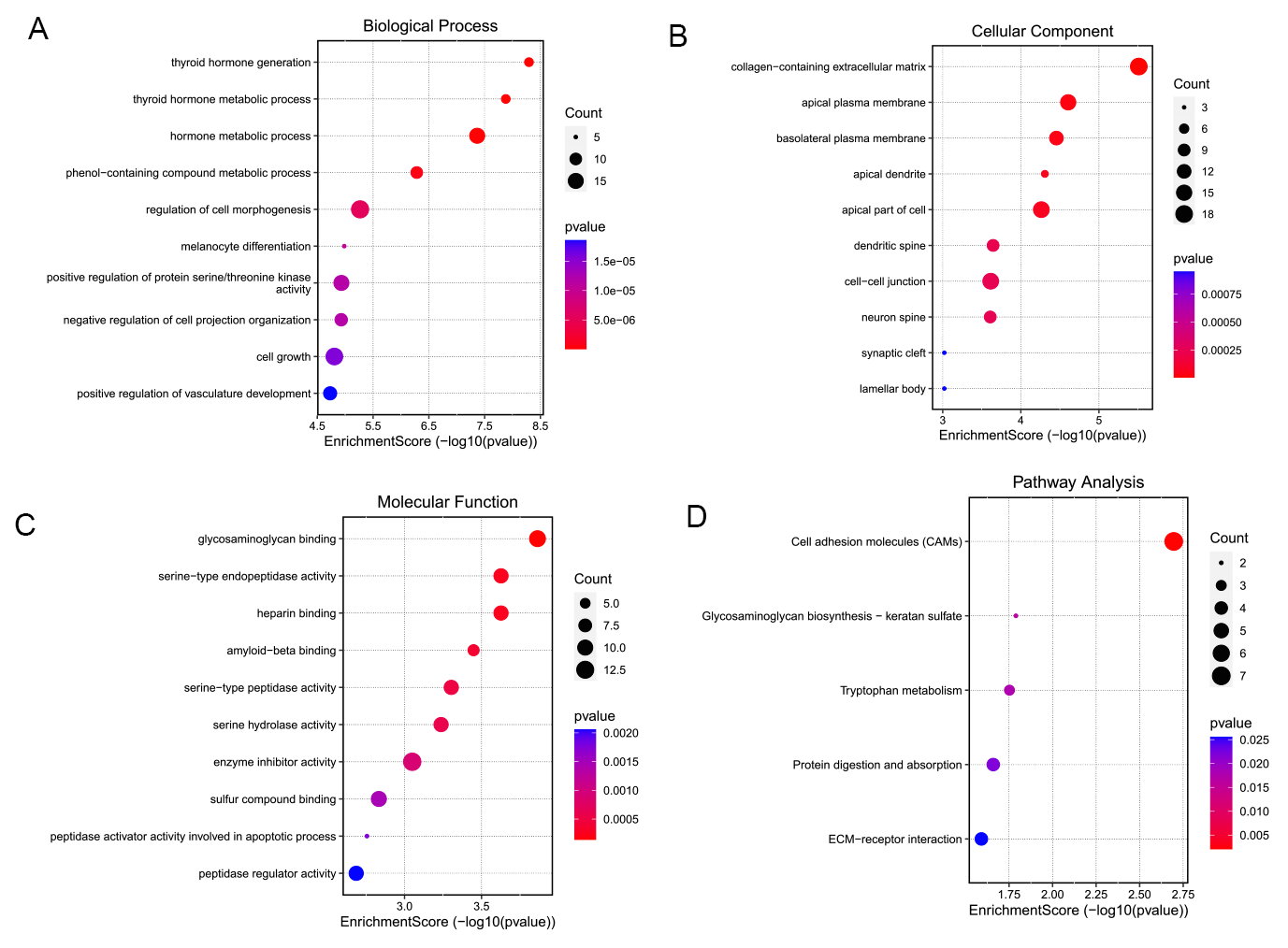

Supplement: Supplementary file 1 [file cancers-14-01353-s001.zip › Figure S4.tif]

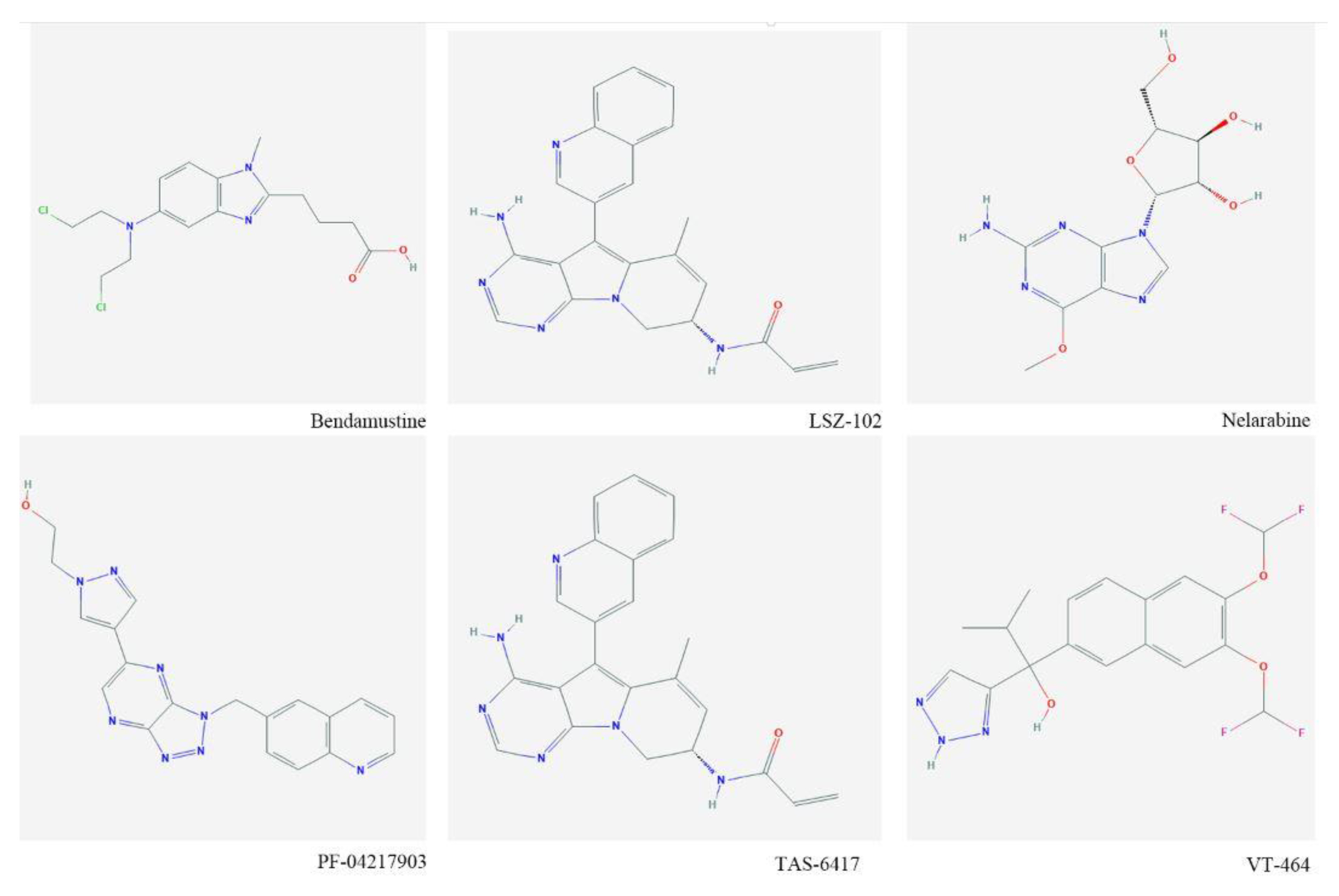

Supplement: Supplementary file 1 [file cancers-14-01353-s001.zip › Figure S5.tif]

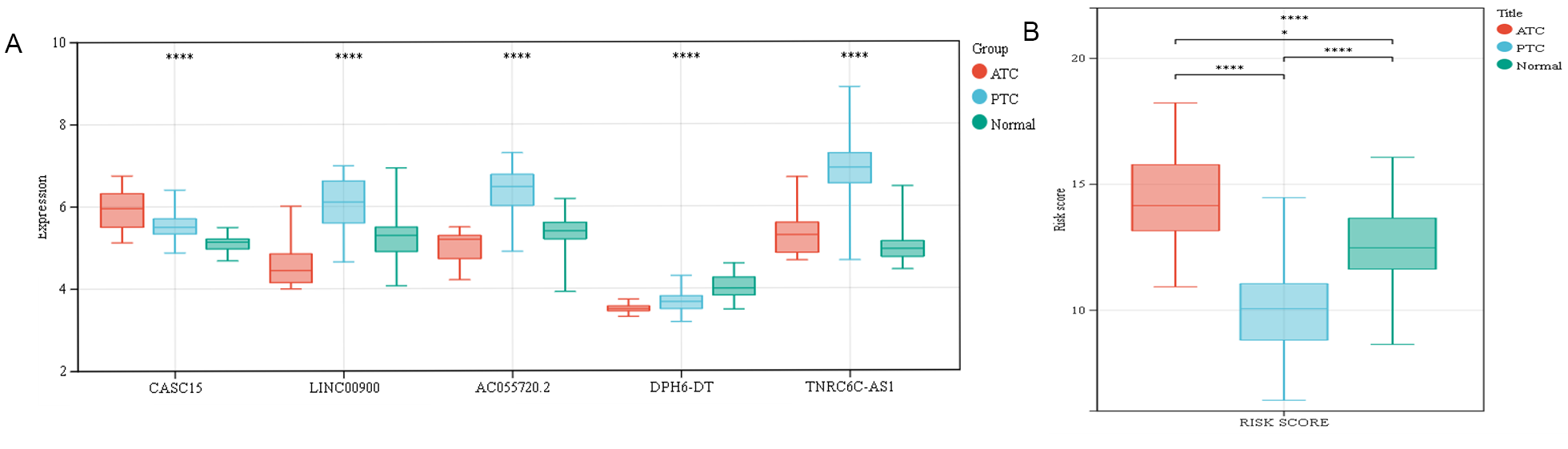

Supplement: Supplementary file 1 [file cancers-14-01353-s001.zip › Figure S6.tif]
